# The influence of resilience and social support on mental health of older adults living in community: a cross-sectional study

**DOI:** 10.1186/s40359-024-01892-6

**Published:** 2024-07-18

**Authors:** Ratchaneekorn Upasen, Weeraphol Saengpanya, Wanida Awae, Preedanan Prasitvej

**Affiliations:** 1 Faculty of Nursing, Chulalongkorn University, Borommaratchachonnanisrisataphat Building, the 11th floor, Rama I road, Bangkok, 10330 Thailand; 2 Faculty of Education, Division of Educational Psychology, Department of Educational Research and Psychology, Thinking, Disposition, and Mental Health – Research Unit (TDMH-RU), Chulalongkorn University, Phayathai road, Pramingkwaun Building, Floor 7, Bangkok, 10330 Thailand; 3 Faculty of Nursing, Princess of Naradhiwas University, Narathiwat Province, 99 moo 8, Khok-Kian Sub-district, Mueang District, 96000 Thailand; 4 Prachomklao College of Nursing, Phetchaburi Province, 203 M.2, Thongchai Subdistrict, Mueang District, Phetchaburi Province 76000 Thailand

**Keywords:** Community, Mental health, Older adult, Psychological resilience, Social support

## Abstract

**Background:**

Mental health is an important fundamental element of health that influences different dimensions of an older individual’s life. There are various factors that affect the mental health of older adults, such as resilience and social support. However, the relationship between various factors and mental health is unclear. Purposes of the study were to investigates whether resilience and social support affect the mental health of older adults and to analyze the structural equation model of mental health among the older adults with resilience as a mediating variable.

**Methods:**

A cross-sectional study was conducted among 964 older adults by multi-stage random sampling. The data were collected using the following questionnaires: Thai Elderly Resilience Scale, Social Support Scale, and Thai Geriatric Mental Health Assessment. Data analysis used descriptive statistics, Pearson’s correlation, and Path analysis.

**Results:**

The older adults had a high level of resilience, social support, and mental health (MH). The best model we obtained demonstrated that social support and resilience had directly statistically significant influences the MH. Social support also had statistically significant indirect effect via resilience to the MH.

**Conclusion:**

Understanding the factors that influence mental health of older adults can be beneficial for health professionals to provide appropriate social support and resilience, including helping the older adult can join with others, be confident in life, have necessities, live with spiritual security, and be able to de-stress and management problems were recommended.

## Background

Aging is an inevitable fact of life. Currently, the Asia-Pacific region is aging more rapidly than any other region in the world, with over 630 million people aged 60 years or over. This represents 60 per cent of the global elderly population. By 2050, this number is projected to increase to 1.3 billion. Women comprise the majority of older adults, constituting up to 61% of those aged 80 years or over. Similar to other countries, Thailand has become an aging society in which the number of adults aged 60 years or over is increasing continuously, accounting for approximately 20% of both the male and female populations, with 5,974,022 men (44.7%) and 7,384,729 women (55.3%) [1]. Many older adults encounter both physical and mental health problems due to several reasons, including physical illnesses, boredom, loneliness, lack of purpose, or having no one to take care of because their children have moved out of the house to find work or migrated to a new location with their own family. These issues can make older adults feel worthless [1, 2]. Moreover, a previous study on the happiness, suffering, and mental health of older adults in the upper north of Thailand reported that most older adults were experiencing illness, were unable to earn an income, and could not find happiness in life. These factors led to changes in their mental health and resulted in more severe mental health issues [2].

Recently, the overall state of mental health in older adults who live in community has changed both positively and negatively [3]. A review on the psychological factors affecting the mental health of older adults, such as a study conducted by MuiJeen [3], found that most older adults had a mean mental health score of 43.92, like that of the general population. This indicates that older adults have the same mental health state and capacity as the general population. Moreover, physical illness, specifically the deterioration of various bodily organs, is the primary factor affecting the mental health of older adults [2, 3]. In addition, changes in social roles, such as retirement, loss of a loved one, and loss of income also affect the elderly’s mental health [4]. A study conducted in Nakhon Ratchasima Province found that 48.3% of the 422 older adults who were leaders of an older adult club had mental health scores similar to that of the general population, 37% had better mental health scores than that of the general population, and only 14% had lower mental health scores than the general population [4]. Hence, the mental health of older adults is another important health issue that requires attention as older adults with low mental health scores require further assistance to improve their mental health [1].

Studies have investigated the factors affecting the mental health of older people, including biological, psychological, social and environmental aspects [2–6]. *In terms of biology*, several factors are related to mental health, including physical illness, abnormal genes, various chemicals in the brain, metabolic abnormalities, congenital diseases, immune, and the side effects of medications. Lifestyle choices and emotional reactions also play significant roles [2]. For instance, a study found that older adults with physical illnesses, congenital diseases, or immune response issues are more likely to have mental health conditions [6, 7]. This study is consistent with findings that chronic illnesses are associated with mental health issues. Essentially, older adults with chronic illnesses tend to have poorer mental health. Additionally, sex and income were found to affect the mental health of older adults [7].

Additionally, a Thai study revealed that marital status and income showed different effects on the mental health of older adults [3]. Moreover, a study conducted by Gray and Thongcharoenchupong [5] found that older adults had different mental health scores depending on factors affecting their mental health, such as age, marital status, education, work, socioeconomic status, overall self-assessment, assessment in daily routine, medical benefits, and exercise. That is, the older adults in the 70–79 age group had lower mental health scores than those in the 60–69 age group. They also found that older adults with a higher education had higher mental health scores than those with lower educational levels. Moreover, older adults with a higher income had better mental health scores. Another Thai study revealed that the elderly who engage in exercise had higher mental health scores than those who do not exercise [5, 6].

*The psychological factors* encompass relationships between individuals, emotional trauma (such as sadness, depression), self-esteem, personal attitudes and beliefs, social, cognitive, and coping skills [2]. For example, a study found that older adults who self-assessed as being healthy had higher mental health scores than those who self-assessed as having health problems. Similarly, older adults who assessed their health positively had higher scores in mental health than those who assessed themselves as having problems with self-care and lacking access to treatment [5]. Another study found that difficulties in coping with stress affect the mental health of older adults [6]. Additionally, previous studies by MaKibbin et al. and Chaosuansreecharoen et al. [8, 9] found that the health of the elderly, along with their self-esteem and happiness, were predictors of mental health. Resilience is another psychological factor that is defined as ability to bounce back to normal life and remain mentally healthy in the face of adverse life event, which consist of the following five components: being able to join with other people, being confident in life, have social support, living with spiritual security, and being able to de-stress and manage problems [10]. Resilience was found to be an influential factor on mental health among the elderly [11–15]. In addition, Tyler et al. [12] found that social support was associated with resilience among Parkinson’s caregivers, including elderly caregivers, in term of increasing their resilience. Resilience also can be a mediator of the relationship between social support and mental health symptoms. Another previous study also has shown that resilience is a mediator of relationship between loneliness and mental health and physical quality of life [12].

*The Social and environmental* factors associated with mental health among older adults include support from friends and colleagues, social status, family dynamics, culture, and the broader environment [2]. For example, a study revealed that older adults who received support had better mental health than those who did not receive any benefits [5]. In addition, previous study by MaKibbin and Chaosuansreecharoen [8, 9] found that the health of the elderly, along with their social relationships and social networks were predictors of mental health. Previous studies mentioned that social support, including family support and resilience also influence the mental health state of older adults [11–15]. In this study, social support refers to perception of the older adult to quality of received support in different forms of provision of assistance to family, including emotional, tangible, and information support [16]. Social support and the perception of health conditions can jointly predict the mental health state of older adults [17]. Additionally, Li et al. [11] found that sources of social support and resilience also affect mental health among older adults. However, only a few studies exist, and these do not provide sufficient knowledge on the factors influencing mental health among older adults in Thailand.

Most of the previous studies have focused on the mental health of older adults living in one province or one region in Thailand; however, this does not represent the overall picture of older adults in Thailand [6, 17]. Identifying the causal factors of elderly mental health conditions will help healthcare professionals understand and recognize mental health issues experienced by older adults. These mental health issues are significant and require supportive care to prevent the development of further problems and their consequences, including low perceived quality of life [7], which can lead to an increase in suicidal thoughts and self-harm. As long as the older adults are unable to adapt to change, mental health problems can become more severe and can even progress to becoming psychiatric disorders. However, many older adults have varying levels of mental health, ranging from low to medium or high, which may depend on various factors.

**Fig. 1:**
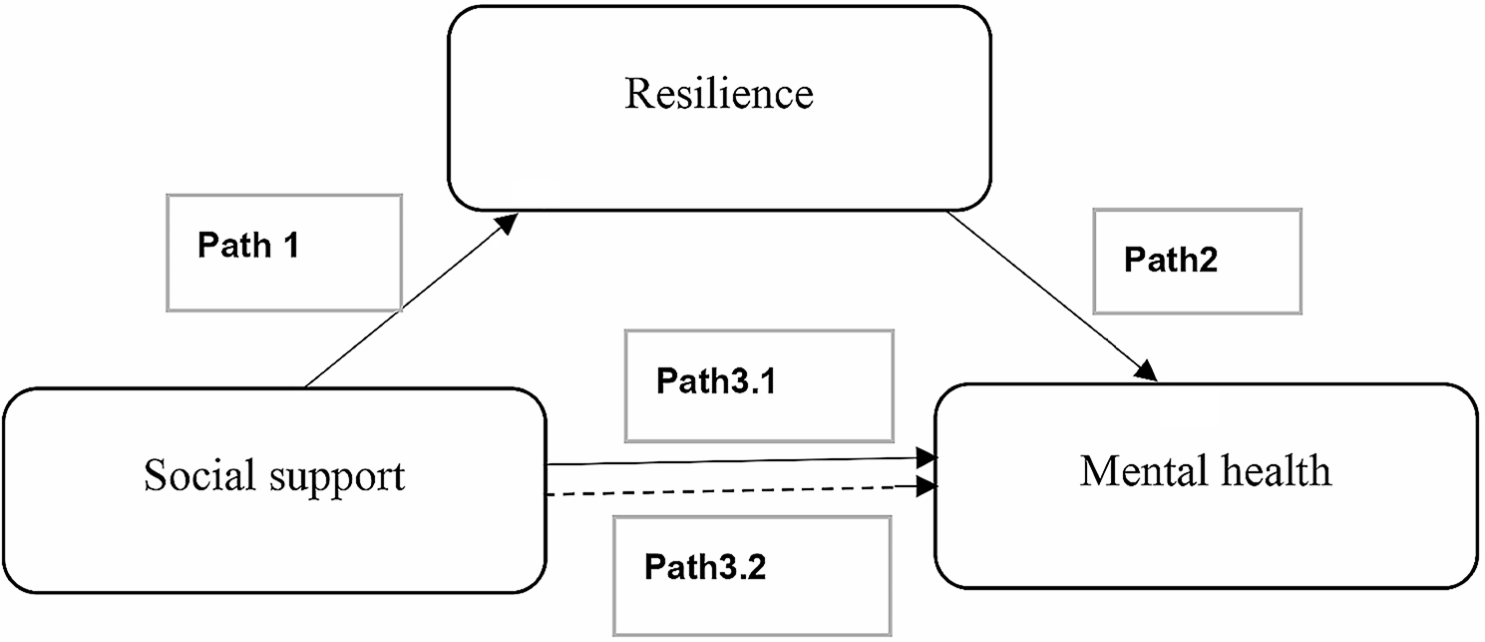
Hypothetical model. Notes: path 1, effect of social support on resilience; path 2, effect of resilience on mental health; path 3.1, total effect of social support on mental health; and path 3.2, direct effect of social support on mental health when resilience is used as a mediator

Thus, the information obtained could be fundamental knowledge that will be useful in planning care for the elderly that is effective, appropriate, and consistent with their needs. Therefore, this study aimed to investigate the level of resilience, social support, and mental health among older adults, to evaluate the effects of resilience and social support on mental health, and to examine the mediating effect of resilience on the relationship between social support and mental health. The researchers also hypothesized that resilience, social support, and mental health of older adults are at a high level, that social support and resilience influence mental health, and that resilience would mediate the relationship between social support and mental health in older adults, as shown in Fig. 1.

## Methods

### Research design and participants

This cross-sectional study was carried out among older adults in Thailand. This method reflects the strength or direction of the relationship between two or more variables that the researcher wants to explore [18, 19]. The population used in this research comprised older Thai adults aged ≥ 60 years, currently living in communities in Thailand. The inclusion criteria were as follows: (a) both male and female with no physical and mental health problem that would affect their participation in the research, such as high blood pressure, tiredness, and fatigue; (b) being able to communicate and listen well; (c) and willing to participate in the research. To assess the physical and mental health of the participants, the vital signs were checked for checking the physical health. For mental health illness, there have no history of mental health illness in OPD card was checked in conjunction with the observation and interview about their physical and mental health. Exclusion criterion was the inability to answer the questionnaire due to more serious health problems. In this study, no participant met the exclusion criterion. We enrolled 964 older adults who met the inclusion criteria. We used multi-stage random sampling and randomly sampling method: table of random sampling in each stage to select the samples from each of the five regions in Thailand, namely the North and Northeast, Central, East, West, and South regions. One province was randomly selected from each region, and one district/community was randomly selected from each province to be used as the research setting.

### Measures

The research instruments in this study were as follows: (1) a demographic questionnaire developed to collect demographic characteristics; (2) Thai Geriatric Mental Health Assessment (The T-GMHA); (3) Social Support Scale, and (4) Thai Elderly Resilience Scale (TERS). The content validity was confirmed by three experts by calculating the content validity index (CVI) of the survey and editing the survey questionnaire according to the CVI and expert recommendations. The three experts consisted of one psychiatrist who is an expert in mental health in older adults. In addition, two experts are advance practice nurses in psychiatric mental health nursing. They are working on research and teaching about mental health in older adults in Thailand. Subsequently, a pilot study was carried out in a group of 50 people with characteristics similar to the real sample in order to check the quality of each item, specifically in terms of the discrimination ability and reliability of each variable indicator, in the developed survey. To examine the quality of each item, discrimination was determined by examining the item–total correlation (r), and the reliability of each item and variable was determined by using the value of Cronbach’s a. When considering the overall instruments, as following:

#### Demographics

Survey items collected demographic data on participants’ gender, age, married status, educational level, religious, monthly income, occupation, exercise, family living, chronic disease, and participation in social activities.

#### Thai geriatric mental health assessment

Mental health also refers to a person’s condition with regard to their psychological and emotional well-being. The research instrument used for measuring the mental health of the older adults, i.e., The T-GMHA (Thai version), developed by Ukaranan et al. [20]. It was developed to be a short version (15 items) for older adults, with reliability obtained by analyzing the Cronbach’s a (α = 0.86). The instrument has four components: mental state (α = 0.54), mental performance (α = 0.78), mental quality (α = 0.81), and support quality (α = 0.84). The overall content validity was equal to 1.

#### Social support scale

Family support in this study is defined as the older adult’s perception to quality of received support in different forms of provision of assistance to family members, including emotional, tangible and information support [16]. Social support scale in this study was adapted from the social support questionnaires according to the concept of social support developed by Schaefer et al. [16] to be appropriate for the context of the older adults who live in community. The items focused on appraisals of quality of the support received from family members. For example, “You received caring from your family”. It differs from the items of a sub-factor of resilience: have social support. The research instrument used for measuring the social support received by older adults, i.e., Social Support Scale (Thai version) developed by Lohasheewa [21] based on the concept of social support reported by Schaefer et al. [16], This instrument consisted of seven items, with reliability obtained by analyzing the Cronbach’s a (α = 0.93). The instrument has two components: emotional support (α = 0.89) and information and tangible support (α = 0.90). The overall content validity was equal to 1.

#### Thai elderly resilience scale

Resilience is defined as the ability to bounce back to normal life and remain mentally healthy in the face of adverse life event, which consists of the following five components: 1) being able to join other people, 2) being confident in life 3) have social support 4) living with spiritual security and 5) being able to de-stress and manage problems [10]. The Thai Elderly Resilience Scale. This scale was developed twice, initial as pre-specific domains and later as specified domains. The pre-specified domains were conceptualized through three source of resilience features based on Grothberg: I AM (Inner strength), I HAVE (External support), and I CAN (Social skills and external skills). The specified domains were synthesized from knowledge and empirical indicators from literature reviews” [10]. This scale focused on exploring ability to do and to have something of older adults. For example, having social support is a sub-factor that based on a source of resilience “I have”. Items focused on asking about ability to have external support of the older adult. The research instrument used for measuring the resilience of the older adults, i.e. TERS (Thai version) developed by Maneerat et al. [22] based on the concept of resilience reported by Grotberg [10], consisted of 19 items, with reliability obtained by analyzing the Cronbach’s a (α = 0.91). The instrument has five components: being able to de-stress and manage problems (α = 0.62), living with spiritual security (α = 0.81), have social support (α = 0.77), being confident in life (α = 0.80), and being able to join other people (α = 0.77). The overall content validity was equal to 1.

### Data collection

After receiving permission to collect data, a research team consisting of researchers, research assistants, and gatekeepers held a meeting to plan the data collection procedure. The data collection process and guidelines for using each type of measurement to collect data as well as the objectives for each type of testing were discussed to check and confirm the understanding of research assistants and coordinators in each province, and, for gatekeepers, to schedule the date, time, and place for data collection. Then, the researchers prepared questionnaires and necessary materials, such as pencils and erasers, sufficient for the sample group, and collected data on the date and time of the appointment. The data collection period was from 18 May 2021 to 17 May 2022.

#### Protecting the participants

Before answering the questionnaire, the physical and mental health status of the participants was assessed. The researcher evaluated the participants by checking vital signs and observing and asking questions about their physical and mental condition. If an older participant exhibited health problems, such as high blood pressure or extreme fatigue to the point of being unable to answer the questionnaire, the researcher provided a break and rechecked their vital signs. In this study, none of the participants had any health problems. Additionally, the participants were informed that they could withdraw from the research at any time without affecting their public health benefits.

During data collection, the researchers continued to provide physical and mental support by ensuring a comfortable environment, such as a breezy, airy, and cool place that was neither too hot nor too cold. They offered drinking water to quench their thirst, listened to them, and facilitated meetings with mental health professionals for counseling if desired by the older adults. Moreover, the researcher provided advice and consultation on various health care matters and coordinated with public health officials responsible for older adult’s care in that district. This ensured that the older adults received timely health services at nearby health facilities.

### Data analysis

A preliminary data analysis was performed to obtain information about the characteristics of the particip3ants and to characterize the variables used in the research using SPSS for Windows version 27. The data were analyzed to answer research questions, which included an analysis of basic statistical values and levels of variables in a structural equation model (SEM) on mental health conditions of the older adults, and an analysis to determine the validity of the SEM of mental health among the older adults. This is an analysis of both direct and indirect influences to verify the coherence of the developed model with the empirical data using the Lisrel Model path analysis technique. Analysis of the SEM examined the coherence between the developed relationship model of older adults’ mental health with empirical data and aimed to analyze the direct and indirect influences of the variables affecting the mental health of the participants according to the aforementioned SEM. For evaluation the model fit, a set of fit indexes were used based on recommended cutoff criteria, including Chi-Square/degree of freedom (χ^2^/df) value should not higher than 2 or 3; a comparative fit index (CFI) and Tucker-Lewis index (TLI) ≥ 0.95, which showed an acceptable fit of the model; the root mean square error of approximation (RMSEA) and standardized root mean square residual (SRMR), where value ≤ 0.05 can be regarded as an appropriate fit and value between 0.05 and 0.08 as an acceptable fit [23]. The analysis of SEM was used by reviewing related documents, concepts, theories, and research to find the relationship model of the participant’s mental health.

### Theoretical framework and variables in the study

According to the accelerated population growth of the older adults worldwide, the World health organization (WHO) emphasize in mental health promotion for older population such as in South-East Asia Region. The promoting mental health policy among older people requires adequate awareness, sufficient human resources and infrastructure, strong psychosocial support, the use of innovative care methods, ongoing research, and reasonable funding. In addition, it is crucial to recognize the factors influencing the mental health of older adults, particularly the impact of resource scarcity to support the older adults, which significantly affects the mental health care of the older population [24]. Additionally, providing a comprehensive mental health action plan is necessary to protect and promote the mental well-being of all citizens [25].

In Thailand, which is facing an aging society, the policy focus on active and healthy aging through 3s (social, strong, and security). There is an emphasis on promoting happiness among older adults through organized activities. The objective is to create a practical model that both promotes mental health and prevents mental health problems among at-risk elderly groups within the community both urban and rural community [26]. In order to align with the policy of raising the level of service for older people, organizations and relevant networks must respond to their needs [27].

Thereby, in this study, the concept of social support [16], resilience [17], and mental health [20] were used. The literature review showed that social support was associated with resilience and mental health state of older adults [11–15]. Resilience was associated with mental health in old age [12–14]. Thus, these variables were selected to investigate how they work together for mental health in older adults. The relationship model of the older adults’ mental health in this study consisted of eleven observable variables of latent variables. The observable variables of latent variable “social support” consisted of two variables, namely emotional support (SS1) and support for information and tangible (SS2). For the observable variables of latent variable “resilience” consisted of five variables, namely, being able to join with other people (RS1), being confident in life (RS2), have social support (RS3), living with spiritual security (RS4), and being able to de-stress and manage problems (RS5). In addition, the observable variables of latent variable “mental health” consisted of four variables, namely, mental state (MH1), mental capacity (MH2), mental quality (MH3), and support quality (MH4).

## Results

### Demographic characteristics of the participants

The researchers collected data from 964 older adults aged ≥ 60 years from representative provinces in the different regions in Thailand. The results of the demographic analysis of participants revealed that most of the participants were female (64.21%), most likely < 65 years of age (34.34%) and followed by aged 66–70 years (28.42%), respectively (M ± SD = 69.59 ± 7.13). Most of them were married (57.88%), followed by widowed (31.22%). Most of them completed primary education (72.07%), were Buddhist (99.07%). Most participants did not have occupations (43.93%), followed by being employed (20.71%). As for physical activity, 39.75% were exercising occasionally (1–2 times/week), followed by having a regular exercise (daily) at 31.84%. Most of the participants were living with family members (offspring) (61.93%). In terms of history of chronic diseases, most had high blood pressure (52.13%), followed by other chronic diseases such as bone diseases, cancer, fatigue (33.67%) and diabetes (28.41%), respectively. The results of the data analysis are shown in Table 1.

**Table 1:** Demographic characteristics of participants (*n* = 964)

Variables	M ± SD or *n* (%)		
**Age** 69.59 ± 7.13 **Gender**			
Men Women	345 619	(35.7) (64.3)	
**Married status**			
Married Single Widow Divorce/separate	558 68 301 37	(57.8) (7.1) (31.3) (3.8)	
**Educational level**			
No Education Primary Education Secondary Education/Vocational certificate Diploma/High Vocational Certificate Bachelor’s Degree or Above	93 694 125 21 30	(9.6) (72.1) (12.9) (2.2) (3.2)	
**Occupation**			
Employee Trader Pensioner No Occupation Other	198 127 37 420 174	(20.8) (13.3) (3.8) (43.9) (18.2)	
**Exercise**			
Regular (Daily) Often (3–4 Times/Week) Some time (1–2 Times/Week) No Exercise	306 154 382 119	(31.8) (16.2) (39.7) (12.3)	
**Family Living**			
Live alone (No Offspring) Live with Family Member (Offspring) Live with Spouse Others	65 597 270 32	(6.7) (61.9) (28.1) (3.3)	
**Total**	**964**	**(100.0)**	
**Chronic Disease (Multi response)**			
High Blood Pressure Diabetes Dyslipidemia Heart Disease Osteoarthritis Other Chronic Diseases (Cancer, Leg Pain.)	466 254 217 62 132 301	(52.1) (28.4) (24.2) (6.9) (14.7) (33.6)	
**Participation in social activities (Multi response)**			
No Participation in Social Activities Participate an Association/School for the Elderly Participate Activities on Important Days with the Community	179 288 598	(19.8) (31.9) (66.3)	

Note. SD = Standard Deviation

### Level of resilience, social support, and mental health of older adults

This study revealed that: (1) The participants had a high level of resilience in total (M ± SD = 58.90 ± 8.12, Min = 29 Max = 74, Skewness = 0.11, and Kurtosis= -0.19). Older adults were able to join other people, living with spiritual security, being confident in life, and being able to de-stress and manage problems at a high level, except for have social support, which was at a moderate level. (2) The participants had a high level of social support in total (M ± SD = 22.02 ± 4.97, Min = 0 Max = 35, Skewness=-0.85, and Kurtosis = 1.31). Participants had emotional support at a moderate level, and the support for information and tangible was at a high level. (3) The participants in this study also had better mental health than the general population (M ± SD = 50.77 ± 6.19, Min = 29 Max = 74, Skewness=-0.16, and Kurtosis= -0.02). The level of mental health, including mental state, mental performance, mental quality, and support quality, was at a better level for the older adults than for the general population in all components. The normality test in Table 2 showed this sample can be defined as having a normal distribution. Normal distribution is a basic assumption underlying the standard use of structural equation modeling is that observations are drawn from a continuous and normal distribution. This assumption is particularly important for maximum likelihood (ML) estimation because the maximum likelihood estimator is derived directly from the expression for the multivariate normal distribution [23]. The details of the level of resilience, social support, and mental health of the participants are shown in Table 2.

**Table 2:** Analysis of the level of resilience, social support, and mental health of the older adults

Variables	M ± SD	Level	Min	Max	Skewness	Kurtosis	Normal distribution
**Resilience**	**58.90 ± 8.12**	**High**	32.00	76.00	0.11	-0.19	Yes
Able to join other people	12.05 ± 2.26	High	2.00	8.00	-0.05	-0.37	Yes
Living with spiritual security	9.90 ± 1.53	High	9.00	20.00	-0.10	-0.30	Yes
Have social support	11.98 ± 2.42	Moderate	4.00	16.00	-0.05	-0.06	Yes
being confident in life	12.61 ± 2.23	High	5.00	16.00	-0.24	-0.19	Yes
Able to de-stress and manage problems	12.37 **±** 1.98	High	4.00	16.00	-0.01	0.22	Yes
**Social Support**	**22.02 ± 4.97**	**High**	0.00	35.00	-0.85	1.31	Yes
Emotional support	9.59 ± 2.20	Moderate	0.00	15.00	-0.94	1.53	Yes
Information and tangible	12.43 ± 3.02	High	0.00	20.00	-0.83	1.08	Yes
**Mental Health**	**50.77 ± 6.19**	**High**	29.00	74.00	-0.16	-0.02	Yes
Mental state	9.67 ± 1.53	High	4.00	13.00	-0.31	-0.05	Yes
Mental performance	12.55 ± 1.97	High	1.94	6.00	20.00	0.01	Yes
Mental quality	13.23 ± 1.96	High	6.00	19.00	-0.23	-0.14	Yes
Support quality	13.27 ± 2.20	High	4.00	20.00	-0.52	0.12	Yes

Note. M = Mean, SD = Standard Deviation, Skewness and Kurtosis range is -2.00 to 2.00

### Correlations analysis of the observable variables in the causal model of older adults’ mental health

This study revealed that all pairs of variables had a statistically significant positive correlation at the 0.01 level with a Pearson correlation coefficient between 0.20 and 0.81, as shown in Table 3. Thus, the data are suitable for further SEM analysis.

**Table 3:** Pearson’s correlation coefficient, mean, and standard deviation of the observable variables in the causal model of mental health of the older adults with resilience as the mediating variable

Pearson’ s correlation *r* )	MH1	MH2	MH3	MH4	RS1	RS2	RS3	RS4	RS5	SS1	SS2
MH1	1.00										
MH2	0.47	1.00									
MH3	0.43	0.53	1.00								
MH4	0.41	0.54	0.47	1.00							
RS1	0.46	0.61	0.55	0.56	1.00						
RS2	0.28	0.40	0.47	0.29	0.38	1.00					
RS3	0.27	0.37	0.50	0.29	0.40	0.81	1.00				
RS4	0.20	0.42	0.29	0.26	0.30	0.43	0.38	1.00			
RS5	0.34	0.47	0.42	0.34	0.50	0.38	0.38	0.39	1.00		
SS1	0.29	0.53	0.40	0.36	0.48	0.40	0.39	0.49	0.69	1.00	
SS2	0.30	0.46	0.49	0.35	0.43	0.58	0.59	0.48	0.55	0.63	1.00
M	12.05	9.90	11.98	12.61	12.37	9.59	12.43	9.67	12.55	13.23	13.27
SD	2.26	1.53	2.42	2.23	1.98	2.20	3.02	1.53	1.97	1.96	2.20

Note. All r values were statistically significant at the 0.01 level. M = mean, SD = standard deviation, MH1 = mental health 1, MH2 = mental health 2, MH3 = mental health 3, MH4 = mental health 4, RS1 = resilience 1, RS2 = resilience 2, RS3 = resilience 3, RS4 = resilience 4, RS5 = resilience 5, SS1 = social support 1, SS2 = social support 2

### The concordance between the theoretical causal model and empirical data

The results of a reconciliation review between the theoretical causal model and the empirical data using the Mplus statistical computer program and robust maximum likelihood revealed that the causal model is inconsistent with the empirical data. Hence, the researchers conducted a model modification to lessen the statistical agreement by requiring some observable variables to be correlated. The results of the model modification resulted in a causal model of mental health among older adults that was consistent with the empirical data. The statistical values indicating the consistency of the model with the empirical data are as follows: χ^2^ (26, *N* = 958) = 26.87, *p* = .41 χ^2^/df = 1.03, *CFI* = 0.99, *TLI* = 0.99, *RMSEA* = 0.006, *SRMR* = 0.018.

### Effect size of the variables affecting the mental health of older adults with resilience used as a mediating variable

**Fig. 2:**
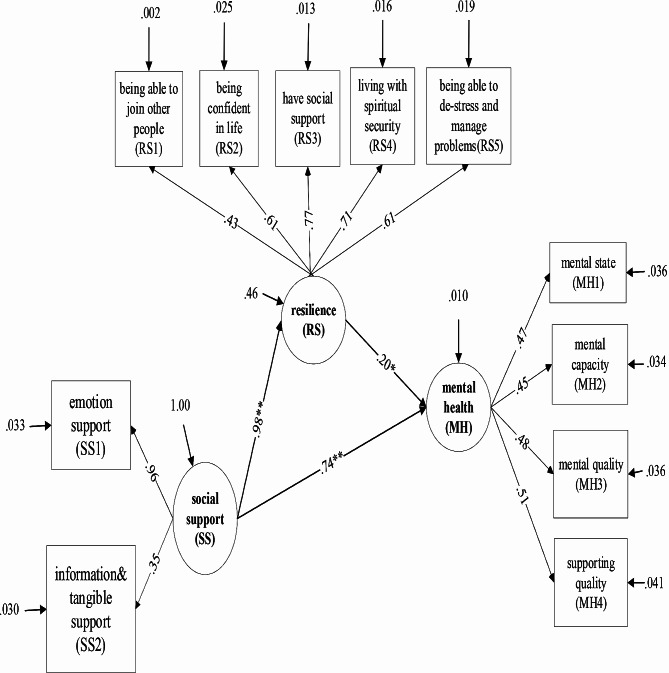
A structural equation model of mental health with resilience as a mediating variable. MH = mental health, MH1 = mental health 1, MH2 = mental health 2, MH3 = mental health 3, MH4 = mental health 4, RS = resilience, RS1 = resilience 1, RS2 = resilience 2, RS3 = resilience 3, RS4 = resilience 4, RS5 = resilience 5, SS = social support, SS1 = social support 1, SS2 = social support 2

The relationship model of mental health among older adults showed that the latent variables that had a statistically significant direct effect at the 0.01 level consisted of the following three paths: (1) SS RS, that is, social support directly affected resilience at a statistically significant level of 0.01 with the size of influence in the form of a standard score (β) = 0.98 (t = 29.15). (2) SS MH, that is, social support directly affected mental health at a statistically significant level of 0.01 with the size of influence in the form of a standard score (β) = 0.74 (t = 3.01). (3) RS MH: that is, resilience directly affected mental health at statistically significant level of 0.05 with the size of influence in the form of a standard score β = 0.20 (t = 2.29). In addition, the causal model of mental health among older adults, there was only one indirect influence that is statistically significant pathway affecting mental health: SS - RS MH. Social support (SS) was described as an endogenous variable that influenced MH through the resilience (RS), which is a mediating variable, where the magnitude of indirect influence in the standard score (β) is equal to β_MH on RS_ x β_RS on SS_ equals 0.20 x 0.98 = 0.20. Details are shown in Table 4; Fig. 2.

**Table 4:** The results of the review of the concordance between the theoretical causal model and empirical data, size of direct influence, indirect influence, and combined influence of variables affecting mental health of older adults

Variables		Resilience (RS)			Mental health (MH)		
		DE	IE	TE	DE	IE	TE
Social support (SS)		0.98**	-	0.98**	0.74**	0.20*	0.94*
Resilience (RS)		-	-	-	0.20*	-	0.20*
Goodness-of-fit indices		R^2^ = 0.87			R^2^ = 0.93		
χ^2^	*df*	*p-value*	χ^2^/df	*CFI*	*TLI*	*RMSEA*	*SRMR*
26.87	26	0.41	1.03	0.99	0.99	0.006	0.018

Note. ** = *p* < .01, * = *p* < .05

χ^2^/df = Chi-Square/degree of freedom, *p* *=* *p*-value, CFI = Comparative Fit Index, TLI = Tucker-Lewis Index, DE = direct effect, IE = indirect effect, TE = total effect, SRMR = Standard Root Mean Residual, RMSEA = Root Mean Square Residual

## Discussions

The findings of this study revealed that older adults had high levels of resilience, social support, and mental health. The best model we obtained demonstrated that social support had a statistically significant with highly direct effect on mental health. Additionally, social support had a statistically significant indirect effect on mental health via resilience. This means that the mental health of older adults was influenced in various ways by support from their families and society, as well as by their resilience. Previous studies that conducted in different contexts and populations suggest that social support improves older adults’ mental health, [13, 31, 32, 33] aligning with this finding. However, other studies have focused solely on social support and its relationship with quality of life [17, 21].

Social support for older adults, especially in Thai contexts, primarily comes from family members such as spouses, children, grandchildren, relatives, and siblings, as well as from the broader society. In Thai culture, families are typically extended, with older adults living with their spouse and children [28]. Younger family members respect and support the older adults, feeling a sense of duty and responsibility toward them. They provide care in various ways, including assisting with daily activities like eating, sleeping, and exercising, as well as managing the environment. These factors contribute to good mental health in older adults [28, 29]. The social support includes three aspects: informational, tangible, and emotional. Informational support involves receiving consistent information, advice, and health care guidelines from family members, especially spouses, relatives, or grandchildren. For tangible support includes assistance with daily routines, financial aid, and other forms of help. Emotional support means receiving attention, love, care, and encouragement from family members [16]. Emotional support is crucial for mental health, helping individuals feel less isolated and better able to cope with stress. However, participants reported receiving only moderate emotional support from their families. Often, children and grandchildren are busy with work or study, leading to insufficient emotional support for the elderly. Future studies should explore ways to improve emotional support for older adults, such as spending time with them, talking to them, and providing encouragement. This could significantly enhance the mental health of older adults.

Meanwhile, the current study found that receiving social support directly influences resilience before affecting the mental health of older adults. This means that when they get social support, older adults will become mentally stronger as a result. They will be able to live with others with confidence and have enough spiritual stability to live, leading to a better MH state. In the same way, the research findings in China found that social support helps older adults face with negative experiences when they have low resilience, resulting in better MH conditions [11]. This previous study highlights the importance of social support, demonstrating that it can buffer the impact of low resilience on mental health. That study investigated sources of social support, and resilience on mental health across all ages (18–85 years old). In contrast, the current study specifically examines older adults who face physical and mental challenges. Furthermore, this study revealed that social support has a strong direct effect on both resilience and mental health, enhancing our understanding of the relationships among these variables and examining their structural relationships.

In addition, this study revealed that older adults had a high level of overall resilience, which significantly impacts their mental health. This indicates that resilience helps older adults manage and reduce the severity of challenging situations. Resilience, which can be developed at any age, aids in building self-esteem and finding appropriate solutions to problems [10]. Meanwhile, resilience can allow the older adults to gain confidence and find ways to solve problems they encounter. The results also showed that improved mental health in older adults enables them to better prevent or mitigate loss or violence in distressing situations and navigate suffering independently, compared to the general population. The older adult also knows how to relax, such as by exercising regularly or participating in community social activities. Based on the results of this study, older adults received support and resources to help them perform daily activities.

A previous study found that resilience mediates the relationship between social support and health-related quality of life among migrant older adults [34]. In contrast, our study shows that resilience mediates the relationship between social support and mental health (MH). Moore et al. [35] similarly found that older adults with low resilience levels may experience poorer mental health. Additionally, our findings align with a previous study indicating that resilient older adults exhibit strong emotional regulation and problem-solving abilities [36]. The resilience of the older adult may be different from that of other age groups due to unique difficulties. Challenges in life that affect resilience include physical deterioration, chronic illness, and exposure to loss. Additionally, changes in social roles and status, income, and self-worth, as well as shifts in population and social structures, and a lack of caregivers, all contribute to these difficulties. Therefore, it is important to promote resilience in the elderly, enabling them to move forward and continue living happily according to their potential.

Moreover, when considering the personal information of the participants, most participants were female, aged 65, and married. These factors are linked to high mental health scores as indicators of healthy aging. They lived with their families, especially spouses and grandchildren, highlighting the importance of family support. Participants received assistance from their children and had counselors or friends for conversation and activities. Additionally, older adults received financial support from welfare funds or elderly allowances and lived in provincial communities of Thailand, which aligns with the findings of Suwanmanee et al. [30], who found that 79.2% of the older adults were mentally healthy. However, the findings of this study differ from that of Muijeen [3], who studied the MH levels of older adults in Pathum Thani province, where it was found that most older adults had MH scores similar to that of an average person (fair). Moreover, studies differ in their findings on whether there was a difference in the MH scores of older adults grouped by age. Gray and Thongcharoenchupong [5] found that older adults had better MH or had higher MH scores than younger people, i.e., most older adults in their 70s to 79s had higher MH scores than those in their 60s to 69s. Similarly, studies such as in Iran found that the average MH score of older adults was mainly high [31, 37]. Moreover, previous research in China reported findings similar to this current study. They showed that the majority of older adults had high overall MH scores [7], but a study in Thailand revealed that the majority of older adults had higher than the general population [3].

In summary, the current study highlights that social support directly affects both resilience and mental health and indirectly strengthens mental health through its impact on resilience. This suggests that the high level of mental health in older adults may arise not only from their resilience but also from receiving strong social support.

### Strength, limitations, and further research

This study has some strengths in methodology. First, there is a large sample that comes from provinces and communities in Thailand. Second, the study collected data from a heterogeneous population. In addition, this study differs from previous studies that focused on examining the relationship between personal characteristics and the mental health of older adults [31] or specifically explored resilience and social support among only the urban elderly [32]. However, we acknowledge some limitations in this study, such as the reliance on self-reported instruments by the participants. This was mitigated by the fact that if the older adults were unclear about certain items, the researchers re-explained the items to the older adults. Besides, “In this study, all subjects are older adults who have physical illness or severe disease such as cancer, heart disease, and others.

For future research should consider to examine relationships between resilience, social support, and mental health in a longitudinal perspective such as research could compare older adults’ mental health at different each age of elderly. In addition, considering the number of subjects (who have severe disease and who do not have severe disease) in each group must be equal or nearest before analysis is required. Moreover, the interaction effect of the different demographic characteristics, including exercise and physical illness of older adults should also be studied. For example, different ages of older adults may have an interaction effect with other demographic variables on the MH.

## Conclusions and implications of the study

This study highlights that older adults with high levels of mental health benefit from social support and resilience as key influencing factors. The relationship model of social support, resilience, and mental health is beneficial for healthcare professionals, including nurses, psychologist when planning care for older adults. These professionals can develop interventions focused on promoting social support, which includes emotional, informational, and tangible support, combined with enhancing resilience among older adults. This approach helps maintain high levels of mental health, encompassing mental state, mental capacity, mental quality, and support quality. The enhancing resilience should include helping the older adult to join with others, be confident in life, have essential resources, live with spiritual security, and be able to de-stress and management problems. These interventions may be helpful for older adults maintain better mental health continuously.

## Data Availability

The data that support the findings of this study are available on request from the corresponding author. The data are not publicly available due to privacy restrictions.

## Abbreviations

MH: Mental health
SEM: Structural equation model
RS: Resilience
SS: Social support
CFI: Comparative fit index
TLI: Tucker-Lewis index
RMSEA: Root mean square error of approximation
SRMR: Standardized root mean square residual
